# The exoskeleton expansion: improving walking and running economy

**DOI:** 10.1186/s12984-020-00663-9

**Published:** 2020-02-19

**Authors:** Gregory S. Sawicki, Owen N. Beck, Inseung Kang, Aaron J. Young

**Affiliations:** 1 The George W. Woodruff School of Mechanical Engineering, Georgia Institute of Technology, Atlanta, GA USA; 2 School of Biological Sciences, Georgia Institute of Technology, Atlanta, GA USA; 3 Institute for Robotics and Intelligent Machines, Georgia Institute of Technology, Atlanta, GA USA

**Keywords:** Wearable robotics, Assistive devices, Metabolic cost, Walk, Run, Energetic, Economy, Augmentation

## Abstract

Since the early 2000s, researchers have been trying to develop lower-limb exoskeletons that augment human mobility by reducing the metabolic cost of walking and running versus without a device. In 2013, researchers finally broke this ‘metabolic cost barrier’. We analyzed the literature through December 2019, and identified 23 studies that demonstrate exoskeleton designs that improved human walking and running economy beyond capable without a device. Here, we reviewed these studies and highlighted key innovations and techniques that enabled these devices to surpass the metabolic cost barrier and steadily improve user walking and running economy from 2013 to nearly 2020. These studies include, physiologically-informed targeting of lower-limb joints; use of off-board actuators to rapidly prototype exoskeleton controllers; mechatronic designs of both active and passive systems; and a renewed focus on human-exoskeleton interface design. Lastly, we highlight emerging trends that we anticipate will further augment wearable-device performance and pose the next grand challenges facing exoskeleton technology for augmenting human mobility.

## Background

### Exoskeletons to augment human walking and running economy: previous predictions and recent milestones

The day that people move about their communities with the assistance of wearable exoskeletons is fast approaching. A decade ago, Ferris predicted that this day would happen by 2024 [1] and Herr foresaw a future where people using exoskeletons to move on natural terrain would be more common than them driving automobiles on concrete roads [2]. Impressively, Ferris and Herr put forth these visions prior to the field achieving the sought-after goal of developing an exoskeleton that breaks the ‘metabolic cost barrier’. That is, a wearable assistive device that alters user limb-joint dynamics, often with the intention of reducing user metabolic cost during natural level-ground walking and running compared to not using a device. When the goal is to reduce effort, metabolic cost is the gold-standard for assessing lower-limb exoskeleton performance since it is an easily attainable, objective measure of effort, and relates closely to overall performance within a given gait mode [3, 4]. For example, reducing ‘exoskeleton’ mass improves user running economy, and in turn running performance [4]. Further, enhanced walking performance is often related to improved walking economy [3] and quality of life [5, 6]. To augment human walking and running performance, researchers seriously began attempting to break the metabolic cost barrier using exoskeletons in the first decade of this century, shortly after the launch of DARPA’s Exoskeletons for Human Performance Augmentation program [7–10].

**Fig. 1:**
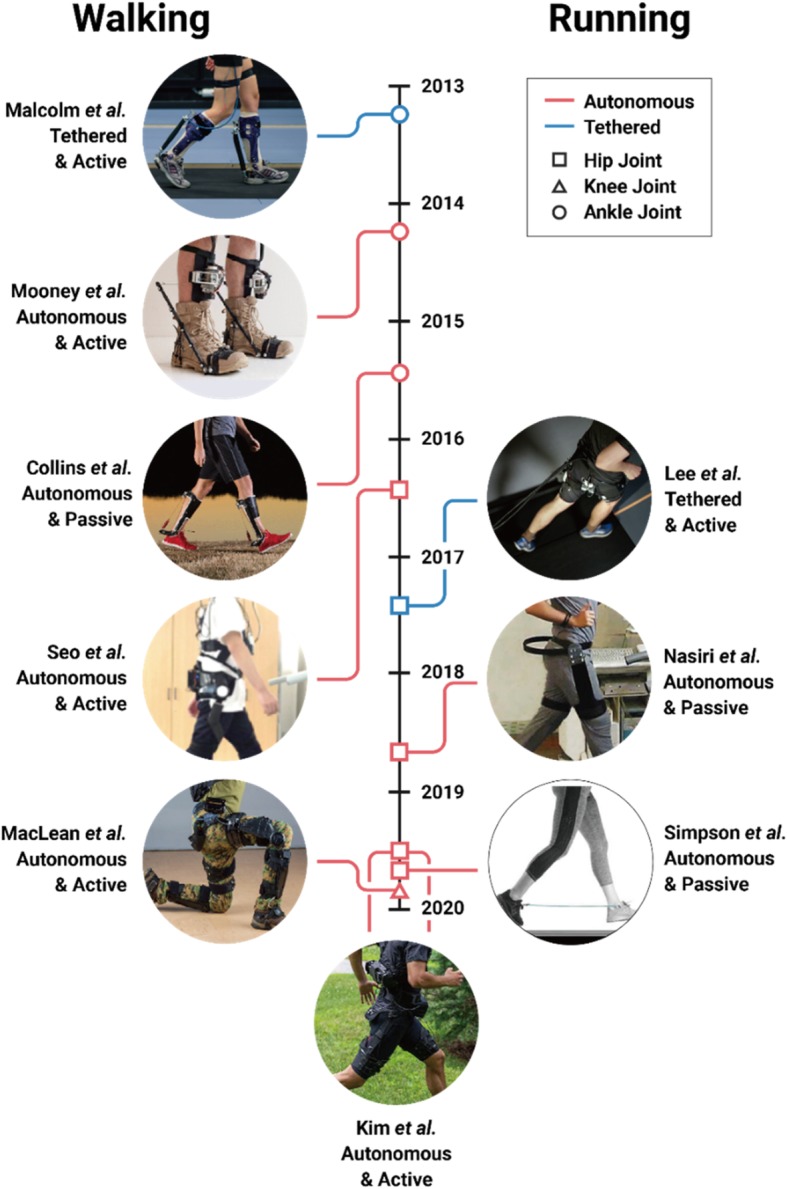
Milestones illustrating the advancement of exoskeleton technology. Tethered (blue) and autonomous (red) exoskeletons assisting at the ankle (circle), knee (triangle), and hip (square) joint to improve healthy, natural walking (left) and running (right) economy versus using no device are shown

It was not until 2013 that an exoskeleton broke the metabolic cost barrier [11]. In that year, Malcolm and colleagues [11] were the first to break the barrier when they developed a tethered active ankle exoskeleton that reduced their participants’ metabolic cost during walking (improved walking economy) by 6% (Fig. 1). In the following 2 years, both autonomous active [12] and passive [13] ankle exoskeletons emerged that also improved human walking economy (Fig. 1). Shortly after those milestones, Lee and colleagues [14] broke running’s metabolic cost barrier using a tethered active hip exoskeleton that improved participants’ running economy by 5% (Fig. 1). Since then, researchers have also developed autonomous active [15, 16] and passive [17, 18] exoskeletons that improve human running economy (Fig. 1).

**Fig. 2:**
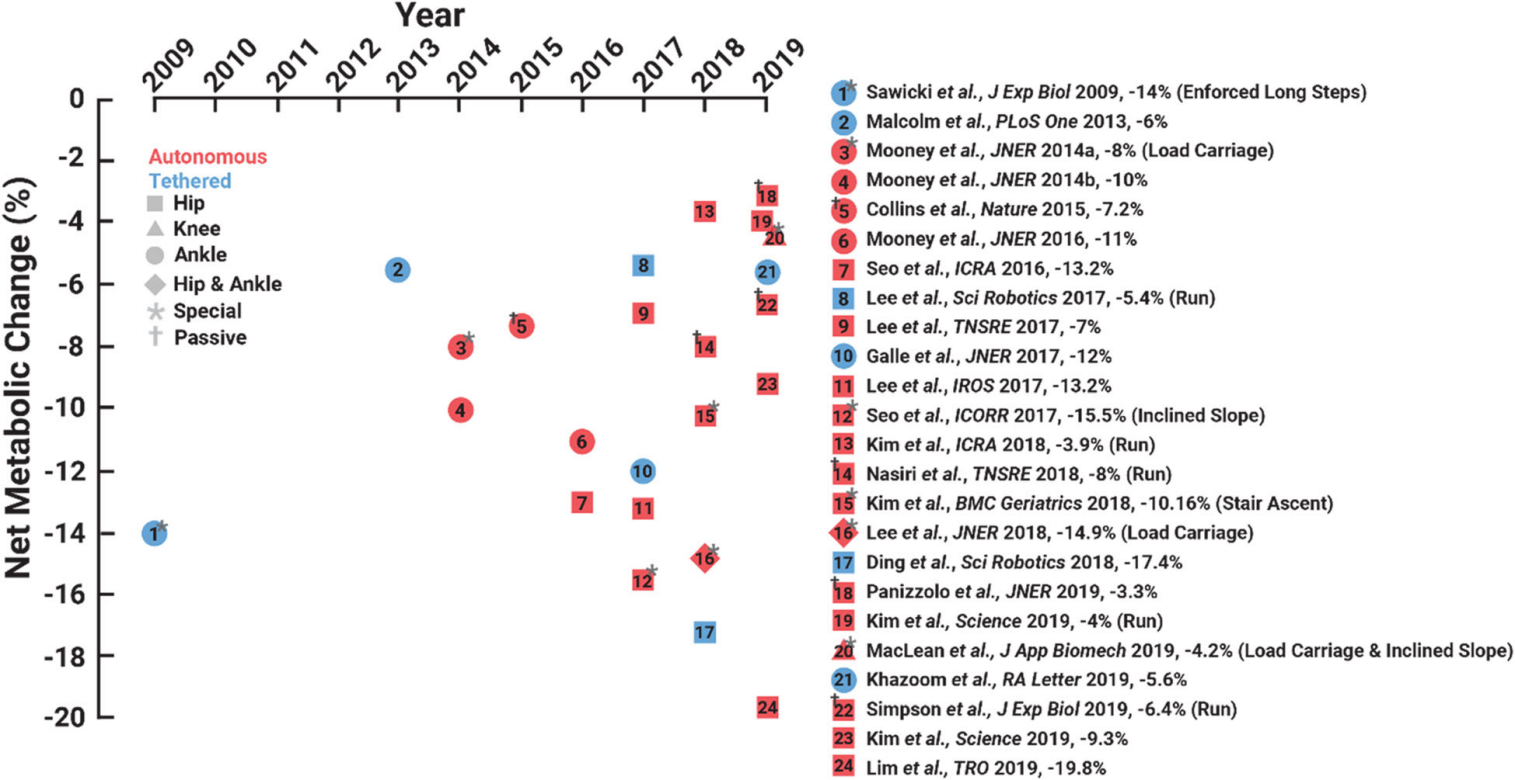
The year that each exoskeleton study was published versus the change in net metabolic cost versus walking or running without using the respective device. Red indicates autonomous and blue indicates a tethered exoskeletons. Different symbols indicate the leg joint(s) that each device directly targets. Asterisk indicates special case and cross indicates a passive exoskeleton

In seven short years, our world went from having zero exoskeletons that could reduce a person’s metabolic cost during walking or running to boasting many such devices (Fig. 2). Continued progress to convert laboratory-constrained exoskeletons to autonomous systems hints at the possibility that exoskeletons may soon expand their reach beyond college campuses and clinics, and improve walking and running economy across more real-world venues. If research and development continues its trajectory, lower-limb exoskeletons will soon augment human walking and running during everyday life – hopefully, fulfilling Ferris’s and Herr’s predictions.

“What a time to be alive” – Aubrey Drake Graham.

## Exoskeleton user performance: insights and trends

To highlight the recent growth of exoskeleton technology, we compiled peer-reviewed publications that reported that an exoskeleton improved user walking or running economy versus without using a device through December 2019. We indexed Web of Science for articles in the English language that included the following topic: (exoskeleton or exosuit or exotendon or assist robot) and (metabolic or energetic or economy) and (walking or running or walk or run). Of the 235 indexed articles, we only included publications that reported that an exoskeleton statistically improved their cohort’s walking and/or running economy versus an experimental no exoskeleton condition. We excluded studies that did not experimentally compare exoskeleton assisted walking or running to a no device condition, choosing to focus on devices that have been shown to break the metabolic cost barrier in the strictest sense. In total, 23 publications satisfied our criteria, and six of these articles improved walking economy during “special” conditions: load carriage [19–21], inclined slope [21, 22], stair ascent [23], and with enforced long steps [24] (Fig. 2 and Table 1). We categorized exoskeletons into a special category, when researchers increased their participant’s metabolic cost above natural level-ground locomotion (e.g. by adding mass to the user’s body), and subsequently used an exoskeleton to reduce the penalized metabolic cost.

**Table 1:** Detailed device specifications for exoskeletons that improved healthy, natural walking, and/or running economy versus using no device

Number	LeadAuthor	Year	Metabolic Reduction (%)	Sample Size	Target Joint(s)	Auto /Tethered	Active /Passive	Walk /Run	Speed (m/s)	Mode	Device Mass (kg)	Note
1	G Sawicki	2009	14	9	Ankle	Tethered	Active	Walk	1.25	Level Ground	2.36	Long Step Lengths
2	P Malcolm	2013	6	8	Ankle	Tethered	Active	Walk	1.38	Level Ground	1.52	
3	L Mooney	2014a	8	7	Ankle	Autonomous	Active	Walk	1.5	Level Ground	4	Load Carry (23 kg)
4	L Mooney	2014b	10	7	Ankle	Autonomous	Active	Walk	1.4	Level Ground	3.6	
5	S Collins	2015	7.2	9	Ankle	Autonomous	Passive	Walk	1.25	Level Ground	0.91	
6	L Mooney	2016	11	6	Ankle	Autonomous	Active	Walk	1.4	Level Ground	3.6	
7	K Seo	2016	13.2	5	Hip	Autonomous	Active	Walk	1.17	Level Ground	2.8	
8	G Lee	2017	5.4	8	Hip	Tethered	Active	Run	2.5	Level Ground	0.81	
9	S Galle	2017	12	10	Ankle	Tethered	Active	Walk	1.25	Level Ground	1.78	
10	Y Lee	2017	13.2	5	Hip	Autonomous	Active	Walk	1.14	Level Ground	2.6	
11	K Seo	2017	15.5	5	Hip	Autonomous	Active	Walk	1.17	Inclined Slope	2.4	5% grade
12	H Lee	2017	7	30	Hip	Autonomous	Active	Walk	1.1	Level Ground	2.8	Elderly
13	R Nasiri	2018	8	10	Hip	Autonomous	Passive	Run	2.5	Level Ground	1.8	
14	S Lee	2018	14.9	7	Hip, Ankle	Autonomous	Active	Walk	1.5	Level Ground	9.3	Load Carry (6.8 kg)
15	Y Ding	2018	17.4	8	Hip	Tethered	Active	Walk	1.25	Level Ground	1.37	
16	J Kim	2018	3.9	8	Hip	Autonomous	Active	Run	2.5	Level Ground	4.7	Hybrid System
17	D Kim	2018	10.16	15	Hip	Autonomous	Active	Walk	N/A	Stair Ascent	2.8	Elderly/128 Steps
18	F Panizzolo	2019	3.3	9	Hip	Autonomous	Passive	Walk	1.1	Level Ground	0.65	Elderly
19	M MacLean	2019	4.2	4	Knee	Autonomous	Active	Walk	0.5	Inclined Slope	8.4	Load Carry (18.1 kg) / 15 deg incline
20	C Simpson	2019	6.4	12	Hip	Autonomous	Passive	Run	2.67	Level Ground	N/A	Ankle Attachment
21	J Kim	2019	9.3	9	Hip	Autonomous	Active	Walk	1.5	Level Ground	5	Hybrid System
22	J Kim	2019	4	9	Hip	Autonomous	Active	Run	2.5	Level Ground	5	Hybrid System
23	B Lim	2019	19.8	6	Hip	Autonomous	Active	Walk	1.11	Level Ground	2.1	
24	C Khazoom	2019	5.6	8	Ankle	Tethered	Active	Walk	1.4	Level Ground	6.2	

Seventeen publications presented improved human walking and/or running economy using an exoskeleton versus without using a device during preferred level-ground conditions: twelve exoskeletons improved walking economy [11–13, 25–33], four improved running economy [14, 15, 17, 18], and one improved both walking and running economy [16] versus using no device (Fig. 2). These studies demonstrate that exoskeletons improved net metabolic cost during walking by 3.3 to 19.8% versus using no device. For context, improving walking economy by 19.8% is equivalent to the change in metabolic cost due to a person shedding a ~ 25 kg rucksack while walking [34]. Moreover, four exoskeletons improved net metabolic cost during running by 3.9 to 8.0% versus the no device condition (Table 1). Theoretically, improving running economy by 8% would enable the world’s fastest marathoner to break the current marathon world record by over 6 min [35] – How about a 1:50 marathon challenge?

We labeled six studies as “special” due to an added metabolic penalty placed on the user such as load carriage [19–21], enforced unnaturally long steps [24], inclined ground slope [21, 22], and/or stair ascent [23] (Fig. 1). Each of these exoskeletons mitigated the negative penalty by reducing metabolic cost. Yet, in some cases [21, 24], the authors also performed a comparison at level ground walking without an added “special” penalty. In these cases, the exoskeleton did not significantly mitigate (and may have increased) metabolic cost. For other “special” cases [19, 22, 23], exoskeletons have achieved a metabolic cost benefit in other relevant studies using the same device [12, 26]. However, in such cases, there were differences in the experimental setup such as the utilized controller, recruited cohort, and testing conditions.

Despite the popular notion that devices with greater power density (e.g.*,* tethered exoskeletons with powerful off-board motors and lightweight interfaces) would reduce user metabolic cost beyond that capable by autonomous devices, to date tethered systems have not improved user walking/running economy beyond that of autonomous systems (t-test: *p* = 0.90) (Fig. 2). Namely, tethered exoskeletons have improved user net metabolic cost during walking by 5.4 to 17.4% and autonomous exoskeletons have improved net metabolic cost during walking by 3.3 to 19.8%. These data are from a variety of devices (Table 1), walking speeds, and control systems, and thus more rigorous comparisons between autonomous and tethered systems may reveal a more stark performance benefit of tethered systems due to their inherently smaller added mass penalty.

Even though distal leg muscles are thought to be more economical/efficient than proximal leg muscles [36, 37], ankle exoskeletons broke the metabolic cost barrier before hip exoskeletons. Perhaps that is because researchers initially targeted the ankles because they yield the greatest positive mechanical power output of any joint [37]. Notably, only one knee exoskeleton has improved walking economy [21] (Fig. 2). Finally, hip exoskeletons (17.4% metabolic reduction for a tethered device and 19.8% for an autonomous device) have numerically improved metabolic cost by more than ankle exoskeletons (12% metabolic reduction for a tethered case and 11% for an autonomous device), perhaps due to the physiological differences between ankle and hip morphology [37, 38] and/or due to the location of the device’s added mass [39].

A closer examination of the subset of exoskeletons that have yielded the greatest metabolic benefit provides insight into the factors that may maximize users’ benefits with future devices. One emerging factor is the exoskeleton controller. There are numerous methods to command [40] and control exoskeleton torque profiles. For example, myoelectric controllers depend on the user’s muscle activity [41, 42] and impedance controllers depend on the user’s joint kinematics [43]. Time-based controllers do not take the state of the user as direct input, and only depend on the resolution offered by the chosen torque versus time parameterization [27, 30, 44]. Recent exoskeleton studies indicate that both magnitude [45, 46] and perhaps more importantly, timing of assistance [11, 47, 48], affect user metabolism. Additionally, time-based controllers have the flexibility to generate a generalized set of assistive torque patterns that can be optimized on the fly and considerably improve walking and running economy over zero-torque conditions [30, 44]. Interestingly, the optimal exoskeleton torque patterns that emerge do not correspond to physiological torques in either their timing or magnitude [14, 44]. But, at least at the ankle, getting the timing right seems paramount, as data from optimized exoskeleton torque patterns show lower variability in the timing versus magnitude of the peak torque across many users [44]. Finally, regarding the magnitude of exoskeleton torque and the net mechanical energy transfer from the device to the user, more is not always better with respect to improving user locomotion economy [13, 27, 44, 46].

## Leading approaches and technologies for advancing exoskeletons

### Exoskeleton testbeds enable systematic, high throughput studies on human physiological response

Tethered exoskeleton testbeds have accelerated device development. In the first decade of the twenty-first century, most exoskeletons were portable, but also cumbersome and limited natural human movement. In addition, these devices were typically designed for one-off, proof of concept demonstrations; not systematic, high-throughput research [49–52]. As researchers began focusing on studies that aimed to understand the user’s physiological response to exoskeleton assistance, a key innovation emerged - the laboratory-based exoskeleton testbed. Rather than placing actuators on the exoskeleton’s end-effector, researchers began placing them off-board and attached them through tethers (e.g.*,* air hoses and Bowden cables) to streamlined exoskeleton end-effectors [45, 53, 54]. This approach enabled researchers to conduct high throughput, systematic studies during treadmill walking and running to determine optimal exoskeleton assistance parameters (e.g.*,* timing and magnitude of mechanical power delivery [27, 55]) for improving walking and running economy. Furthermore, the high-performance motors on recent tethered exoskeleton testbeds have relatively high torque control bandwidth that can be leveraged to render the dynamics of existing or novel design concepts [43, 56]. Testing multiple concepts prior to the final device development could enable researchers to quickly diagnose the independent effects of design parameters on current products and test novel ideas [57]. Thus, we reason that exoskeleton testbeds have progressed exoskeleton technology by enabling researchers to optimize a high number of device parameters [58], test new ideas, and then iterate designs without having to build one-off prototypes.

### Embedding ‘smart mechanics’ into passive exoskeletons provides an alternative to fully powered designs

Laboratory-based exoskeletons are moving into the real-world through the use of small, transportable energy supplies [59] and/or by harvesting mechanical energy to power the device [60]. Despite these improvements, another way to circumnavigate the burden of lugging around bulky energy sources is by developing passive exoskeletons [13, 17, 18, 31]. Passive exoskeletons have been able to assist the user by storing and subsequently returning mechanical energy to the user without injecting net positive mechanical work. Passive exoskeletons are typically cheaper and lighter than active devices (e.g.*,* Collins et al.’s ankle exoskeleton is 400 g [13]) and, like active devices, are hypothesized to primarily improve walking and running economy by reducing active muscle volume [61]. However, due to their simplified designs, passive exoskeletons are in some ways less adaptable than powered devices. Passive devices can only offer fixed mechanical properties that are at best only switchable between locomotion bouts. Thus, while passive systems may be adequate for providing assistance during stereotyped locomotion tasks such as running on a track or hiking downhill at fixed speed, they may not be able to handle variable conditions. On the other hand, active devices offer the opportunity to apply any generic torque-time profile, but require bulky motors and/or gears that need a significant source of power to do so. Thus, combining features from active and passive exoskeletons to create a new class of pseudo-passive (or semi-active) devices may yield a promising future direction for exoskeleton technology [59]. For example, rather than continuously modulating the assistance torque profile, a pseudo-passive device might inject small amounts of power to change the mechanical properties of an underlying passive structure during periods when it is unloaded [62]. The pseudo-passive approach likely benefits from the streamlined structural design (e.g.*,* small motors) and adaptability that requires only small amounts of energy input (e.g.*,* small batteries).

### Providing comfort at the human-exoskeleton interface

Regardless of active or passive exoskeleton design, researchers struggle to effectively and comfortably interface exoskeletons to the human body [63]. That is primarily due to the human body having multiple degrees of freedom, deforming tissues, and sensitive points of pressure. Accordingly, many researchers utilize custom orthotic fabrication techniques [46, 64, 65], and/or malleable textiles (commonly referred to as exo-suits) [16, 66–68] to tackle this challenge. Textile-based exoskeletons may be superior to traditional rigid exoskeletons due to their lower mass, improved comfort, fewer kinematic restrictions, and better translation to practical-use [16, 67, 68]. Reaffirming soft technology, the tethered exoskeleton that best improves walking economy versus not using a device is currently an exoskeleton with a soft, malleable user-device interface [67] (Fig. 2).

### Exoskeleton controllers using artificial intelligence and on-line optimization to adapt to both user and environment may facilitate the transition to ‘real-world’ functionality

Researchers are also developing smart controllers that constantly update exoskeleton characteristics to optimize user walking and running economy. This is exemplified by Zhang and colleagues [44], who developed a controller that rapidly estimates metabolic profiles and adjusts ankle exoskeleton torque profiles to optimize human walking and running economy. We foresee smart controllers enabling exoskeletons to move beyond conventional fixed assistance parameters, and steering user physiology in-a-closed-loop with the device to maintain optimal exoskeleton assistance across conditions [30, 69]. Since measuring metabolic cost throughout everyday life is unrealistic, future exoskeletons may incorporate embedded wearable sensors (e.g.*,* electromyography surface electrodes, pulse oximetry units, and/or low-profile ultrasonography probes) that inform the controller of the user’s current physiological state [70, 71] and thereby enable continuous optimizing of device assistance [20, 72, 73] to minimize the user’s estimated metabolic cost.

At a high level of control, researchers are using techniques to detect user intent, environmental parameters, and optimize exoskeleton assistance across multiple tasks [15, 16, 68, 74, 75]. An early version of this techniques paradigm was implementing proportional myoelectric control into exoskeletons [76–78]. This strategy directly modulates exoskeleton torque based on the timing and magnitude of a targeted muscle’s activity, which can adapt the device to the users changing biomechanics. However, this strategy has yielded mixed results [42, 79, 80] and is challenging to effectively use due to quick adaptations that occur to accommodate various tasks as well as slower changes that occur due to learning the device [41]. Scientists have made exciting advances using machine learning and artificial intelligence techniques to fuse information from both sensors on the user and device to better merge the user and exoskeleton [81, 82], but these techniques have not yet been commercially translated to exoskeleton technology to the authors’ knowledge. These strategies have the potential to enable exoskeletons to discern user locomotion states (such as running, walking, descending ramps, and ascending stairs) and alter device parameters to meet the respective task demands.

## Conclusion

### Closing remarks and vision for the future of exoskeleton technology

In the near-term, we predict that the exoskeleton expansion will break researchers out of laboratory confinement. Doing so will enable studies that directly address how exoskeleton-assistance affects real-world walking and running performance without relying on extrapolated laboratory-based findings. By escaping the laboratory, we expect that exoskeleton technology will expand beyond improving human walking and running economy over the next decade and begin optimizing other aspects of locomotor performance that influence day-to-day mobility in natural environments. To list a few grand-challenges, exoskeletons may begin to augment user stability, agility, and robustness of gait. For example, exoskeletons may make users,

· More stable by modulating the sensorimotor response of their neuromuscular system to perturbations [83–85].
· More agile and faster by increasing the relative force capacity of their muscles [86].
· More robust by dissipating mechanical energy to prevent injury during high impact activities like rapid cutting maneuvers or falling from extreme heights [87].

To make these leaps, engineers will need to continue to improve exoskeleton technology, physiologists will need to refine the evaluation of human performance, clinicians will need to consider how exoskeletons can further rehabilitation interventions, psychologists will need to better understand how user’s interact with and embody exoskeletons, designers will need to account for exoskeletons in space planning, and healthcare professionals may need to update their exercise recommendations to account for the use of exoskeletons. Combined, these efforts will help establish a ‘map’ that can be continuously updated to help navigate the interaction between human, machine, and environment. Such guidelines will set the stage for exoskeletons that operate in symbiosis with the user to blur lines between human and machine. Closing the loop between exoskeleton hardware, software, and the user’s biological systems (e.g.*,* both musculoskeletal and neural tissues) will enable a new class of devices capable of steering human neuromechanical structure and function over both short and long timescales during walking and running. On the shortest of time scales, exoskeletons that have access to body state information have the potential to modify sensory feedback from mechanoreceptors and augment dynamic balance. On the longest of timescales, exoskeletons that have access to biomarkers indicating tissue degradation [88] could modify external loads to shape the material properties of connective tissues and maintain homeostasis.

Until then, we focus our attention on the ability of exoskeletons to improve human walking and running economy. So far, 17 studies have reported that exoskeletons improve natural human walking and running economy (Fig. 2). As these devices evolve and become more available for public use, they will not only continue to improve walking and running economy of young adults, but they will also augment elite athlete performance, allow older adults to keep up with their kinfolk, enable people with disability to outpace their peers, and take explorers deeper into the wilderness.

## Data Availability

Not applicable.
